# Maintenance Therapy With Everolimus for Subependymal Giant Cell Astrocytoma in Patients With Tuberous Sclerosis – Final Results From the EMINENTS Study

**DOI:** 10.3389/fneur.2021.581102

**Published:** 2021-04-09

**Authors:** Katarzyna Bobeff, Karolina Krajewska, Dobromila Baranska, Katarzyna Kotulska, Sergiusz Jozwiak, Wojciech Mlynarski, Joanna Trelinska

**Affiliations:** ^1^Department of Pediatrics, Oncology and Hematology, Medical University of Lodz, Lodz, Poland; ^2^Department of Pediatric Radiology, Medical University Hospital, Lodz, Poland; ^3^Department of Neurology & Epileptology and Pediatric Rehabilitation, The Children's Memorial Health Institute, Warsaw, Poland; ^4^Department of Child Neurology, Medical University of Warsaw, Warsaw, Poland

**Keywords:** everolimus, MTOR inhibitor, maintenance therapy, subependymal giant cell astrocytoma, tuberous sclerosis

## Abstract

The aim of this EMINENTS prospective, single-center, open-label, single-arm study was to evaluate the cumulative efficacy and safety of reduced doses of everolimus (maintenance therapy) in patients with tuberous sclerosis and subependymal giant cell astrocytoma (SEGA).

**Methods:** The trial included 15 patients who had undergone at least 12 months of treatment with a standard everolimus dose. The dose of everolimus was reduced to three times a week, with a daily dose as in standard regimen. Data of 14 patients were analyzed. SEGA volume (SV) was evaluated at study entry and subsequent time points by an experienced radiologist. Adverse events (AEs) noted during maintenance therapy were compared to the AEs of standard dose period.

**Results:** Patients were followed over a mean duration 58.37 months (95%CI: 45.95–70.78). The differences in SEGA volume between subsequent time points (0, 3, 6,12, 18, 24, 36, 48, and 60 months) were not statistically significant (*p* = 0.16). At the end of the study, 7 out of 10 patients had stable SEGA volume. No clinical symptoms of progression were observed in any patients. No patient or tumor-related risk factors of progression were identified. Regarding AEs, infections (stomatitis, bronchitis, diarrhea) and laboratory abnormalities (neutropenia, anemia, hyperglycemia) occurred less frequently during maintenance therapy compared to the standard dose regimen.

**Conclusions:** Final results from EMINENTS study confirm that maintenance therapy with everolimus might represent a rational therapeutic option for patients TSC and SEGA after effective full dose treatment. It could be an option for patients who experienced everolimus-related AEs, instead of discontinuation of therapy. Careful evaluation of possible progression, especially concerning first six months of maintenance therapy should be advised.

**Clinical Trial Registration:** www.drks.de, identifier DRKS00005584.

## Introduction

Tuberous sclerosis (TSC) is an autosomal dominant genetic disorder in which mutation of *TSC1* or *TSC2* genes leads to increased activation of the mammalian target of rapamycin (MTOR) pathway. This results in the growth of benign tumors in multiple organs, including the brain, where the most severe clinical manifestation of TSC is the subependymal giant cell astrocytoma (SEGA). SEGAs develop in up to 20% of patients with TSC and when growing, may cause obstruction of cerebrospinal fluid flow leading to hydrocephalus. Currently, the recommended treatment options for SEGA associated with TSC are surgical resection or MTOR inhibitor (1).

Everolimus is an MTOR inhibitor, which has been recently approved in the United States by the Food and Drug Administration and in Europe by the European Medicines Agency for treatment of patients with TSC-related SEGA who require therapeutic intervention, but whose tumors cannot be curatively resected (2). Everolimus has recently demonstrated therapeutic efficacy and safety in patients with TSC in a number of trials (3, 4).

Evidence suggests that patients with TSC may require long-term treatment with MTOR inhibitors (5, 6). Some reports indicate that SEGAs may grow back after the cessation of MTOR inhibitor therapy (7) and the optimal duration of treatment with an MTOR inhibitor has yet to be determined: such treatment may be life-long. Therefore, safety issues connected to everolimus-related side effects must be taken into consideration. Although the short-term side effects related to everolimus therapy are generally considered acceptable, life-threatening events have also been reported (8). In addition, the long-term side effects are less known and require further research.

Treatment with everolimus results in a rapid initial reduction in SEGA volume, followed by a phase of slower reduction or stabilization of tumor size (9). However, once SEGA has been stabilized with MTOR inhibitor, it can be possible to reduce the dose of the drug in order to minimize any long-term adverse effects of the therapy. In 2014, Wheless et al. (2) proposed an algorithm for dose reduction intended to minimize the adverse effects of MTOR inhibitor therapy for SEGA cases that are stable or decreasing in size. This algorithm was tested for the first time in a clinical setting as part of the first results of the EMINENTS (Everolimus MaINtENance Therapy in SEGA) study published in 2017. The study followed 10 patients on a reduced dose of everolimus over at least 12 months (10). The recruitment was finished in October 2017, and the results of the subsequent ≥24-month analysis are presented herein.

## Patients and Methods

### Study Design

The design of the prospective, a single-center, open-label, single-arm study has been described in detail previously (10). The trial enrolled 15 patients who had undergone at least 12 months of treatment based on a standard everolimus dose. The recruitment was performed between December 19, 2013 and October 25, 2017, and the follow-up continued until January 3, 2020.

The study was approved by the Bioethics Committee of the Medical University of Lodz (# RNN/315/15/KE) and the study was registered in the German Clinical Trials Register (DRKS) (ID: DRKS00005584, http://apps.who.int/trialsearch/). It was conducted in compliance with good clinical practice guidelines and under the principles of the Declaration of Helsinki. All patients were treated at the Department of Pediatrics, Oncology and Hematology, Medical University of Lodz, Poland.

The primary aim of the study was to determine the proportion of patients with stable SEGA volume during reduced-dose everolimus treatment. Stabilization was defined as follows: no changes in the total volume of all SEGAs or an increase <50% relative to study entry or an increase to a volume not exceeding that observed before the start of standard everolimus treatment; no new lesions of 1 cm in diameter and no new hydrocephalus. The secondary objective included a safety profile of maintenance therapy with a comparison to standard everolimus therapy.

### Patients

The patients were recruited from the whole of country. Following recruitment, they were treated and evaluated by an experienced team of pediatric oncologists at the study center. The inclusion criteria were as follows: patients with definite diagnosis of TSC with SEGA; previous treatment with everolimus at a standard dose for a minimum 12 months and a maximum 24 months, resulting in stabilization or reduction of SEGA volume; no signs of increased intracranial pressure and no hydrocephalus observed in brain MRI during evaluation prior to enrolment; signed informed consent by both the patient's parents, and the patients assent for participation in the trial.

### Treatment

The standard treatment protocol for everolimus therapy consisted of oral everolimus administration once daily at the same time every day, consistently either with or without food. Dosing was titrated to attain trough concentrations of 5 to 15 ng/ml. It is described in details in a previous publication (10). After at least 12 months of standard treatment in the group of patients demonstrating reduction or stabilization of SEGA volume, the treatment regimen was changed to everolimus three times a week (Monday, Wednesday, Friday) with the same daily dose (maintenance therapy). Everolimus (Votubia, Novartis, Germany) was provided by Polish National Health Fund for patients with TSC and SEGA diagnosis.

### Evaluation of SEGA Volume

Magnetic resonance imaging (MRI) was conducted before the introduction of standard dose everolimus therapy, at the time of study entry, and then after 3, 6, 12, 18, 24 months of maintenance therapy, and once a year thereafter. All patients were examined using a 1.5-Tesla MRI scanner (Toshiba Medical System, Otawara, Japan) with a standardized protocol for brain examination. Measurements were obtained on enhanced T1-weighted images in three perpendicular planes.

Two methods of SEGA volume assessment were used. All scans were assessed by the same radiologist with 10 years' experience in brain MRI evaluation, who was blinded to the clinical history of the patients (standard dose treatment vs. maintenance therapy). SEGA volume was approximated as an ellipsoid, using the formula: 0.52 x a x b x c, where a, b, c are the maximum dimensions of the tumor measured in the axial, sagittal and coronal planes measured on MRI scans. To avoid potential overestimation of volume due to the ellipsoid approximation, a semi-automated method of volume measurement was also applied using ITK-SNAP software. These have been described in detail in previous publications (10, 11). Since a strong positive correlation was found between the manual and automatic methods of comparing tumor volumes at the same time points (*r* = 0.8925, *p* < 0.0001), only evaluations of the SEGA volume performed by the radiologist were incorporated in further analyses.

### Safety Profile Assessment

The patients were clinically evaluated once per month for the initial 6 months and every 3 months thereafter. All clinical symptoms that occurred during therapy were recorded. Laboratory studies were performed at each study visit; these included complete blood count, fasting lipid profile, glucose level, liver and kidney function tests. Adverse events (AEs) were assessed with the use of the Common Terminology Criteria for Adverse Events (version 4.0) (12). The most severe grade of each AEs per patient per year were recorded. The number of patients with reported AEs during maintenance therapy per year were compared with those observed during standard everolimus therapy, and were statistically evaluated. Everolimus whole blood trough concentration was assessed at each study visit using ultra performance liquid chromatography/tandem mass spectrometry (UPLC/MS/MS) as described previously (13).

### Statistical Analysis

The comparison between multiple groups was performed with the analysis of variance (ANOVA); if ANOVA yielded a significant difference, this was followed by between-group comparisons with the Unequal HSD *post hoc* test. Repeated measures ANOVA was used to evaluate time-dependent changes in tumor size. Mauchly's sphericity test was used to assess the assumption of data sphericity; comparisons that showed significant sphericity were subjected to the Greenhouse–Geisser correction, with an Unequal HSD *post hoc* test if needed. Cochrane's Q-test was applied to evaluate differences in global test for repeated measures with nominal data. Scores with p-levels < 0.05 were regarded as significant. Statistica version 13 (Dell Software) was used for statistical analysis.

## Results

**Figure 1:**
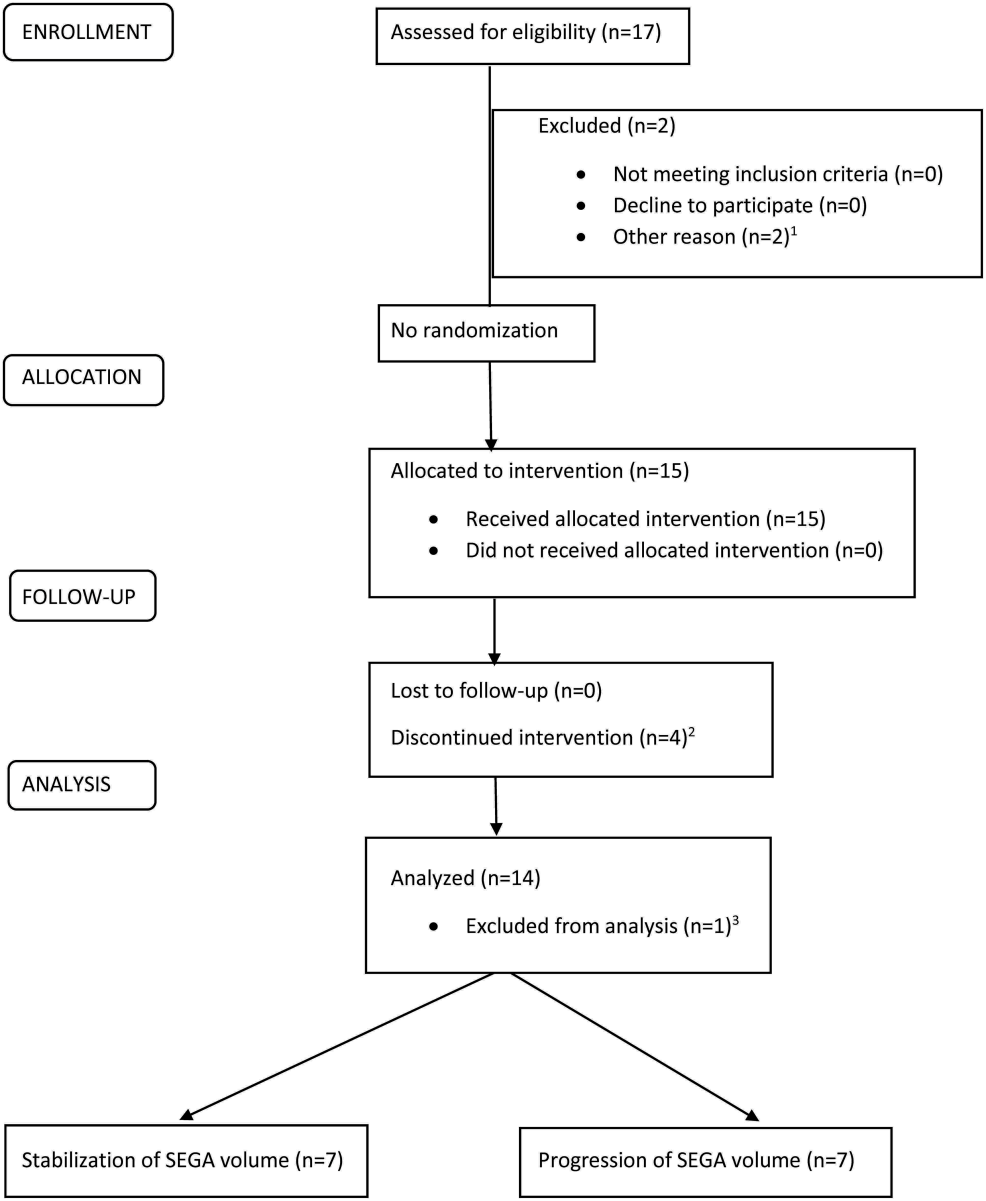
CONSORT flowchart for a single-arm, open-label, prospective intervention to evaluate the efficacy and safety of maintenance therapy with everolimus in patients with TSC and SEGA.

Between December 2013 and November 2017, a total of 15 patients were enrolled into the study; however, one patient was excluded from the analysis due to diagnosis of malignant brain tumor (gliosarcoma grade IV acc. WHO classification) in SEGA location after 4 years of everolimus treatment (after 6 months on maintenance dose). Therefore, only 14 patients were included in the data analysis (Figure 1). The clinical characteristics of study group are presented in Table 1.

**Table 1:** The clinical characteristics of the patients.

**Characteristic**	**Mean (95%CI) or Number (%)**
Age (years) at study entry	13.82 (11.35–16.29)
Full-dose treatment duration (months)	14.48 (11.63–17.34)
Maintenance therapy duration (months)	58.37 (45.95–70.78)
Sex: male	9/14 (64%)
Female	5/14 (36%)
**TSC mutation status**
•*TSC1*	4/14 (29%)
•*TSC2*	8/14 (57%)
•No mutation identified	2/14 (14%)
**Skin lesions**
•Facial angiofibroma	11/14 (79%)
•Fibrous cephalic plaque	3/14 (21%)
•Hypomelanotic macules	13/14 (93%)
•Shagreen patch	10/14 (71%)
**Other features**
•Kidney angiomyolipomas	8/14 (57%)
•Multiple renal cysts	4/14 (29%)
•Cardiac rhabdomyoma	5/14 (36%)
•Retinal hamartomas	4/14 (29%)
TSC-associated neuropsychiatric disorders	10/14 (71%)
Epilepsy	11/14 (79%)
SEGA volume (SV) before treatment (cm^3^)	2.1 (1.1 −3.1)
SEGA volume at study entry (cm^3^)	0.84 (0.44– 1.23)
Percentage of SV at study entry compared to pretreatment (%)	51.47 (32.81– 70.14)
Everolimus dose at study entry (mg/m^2^/week)	41 (34.22–47.77)
Everolimus dose at study end (mg/m^2^/week)	15.37 (12.82–17.91)
Everolimus concentration at study entry (ng/ml)	8.32 (6.58– 10.05)
Everolimus concentration during the study (ng/ml)	2.65 (2.1– 3.19)

The mean duration of maintenance therapy was 58.37 months (95%CI: 45.95–70.78). The mean everolimus dose was 41 mg/m^2^/week (95%CI: 34.22–47.77) at study entrance and 15.37 mg/m^2^/week (95%CI: 12.82–17.91) at study end. The mean everolimus concentration during the study was 2.65 ng/ml (95%CI: 2.1–3.19) (Table 1).

### Tumor Volume Evaluation

The mean SEGA volumes (SV) before and during the study are presented in Supplementary Table 1. The differences in SV between subsequent time points (0, 3, 6,12, 18, 24, 36, 48, and 60 months) were not statistically significant (*p* = 0.16) in the ANOVA repeated measures test.

Throughout the whole dataset, pretreatment SEGA size differed from that measured during the treatment period (*p* = 0.003). Pairwise comparisons between 0, 3, 6, 12, 18, 24, 36, 48, and 60 months and pretreatment values were significant (all p-levels < 0.0002).

During maintenance therapy, an increase in volume of 0.56 cm^3^ per year was observed during the first six months and 0.14 cm^3^ per year in the following 6 months. Further stabilization, i.e., an increase in volume < 0.07 cm^3^ per year, was observed over subsequent months; however, a 0.14 cm^3^ per year increase was observed in the third year.

The lowest proportion of patients with stable SV were observed at the time points 12 and 18 months (62%). This proportion then increased at time points: 36 (83%) and 48 months (82%) – Supplementary Table 1. At the end of the study, seven out of ten patients had stable SV.

**Figure 2:**
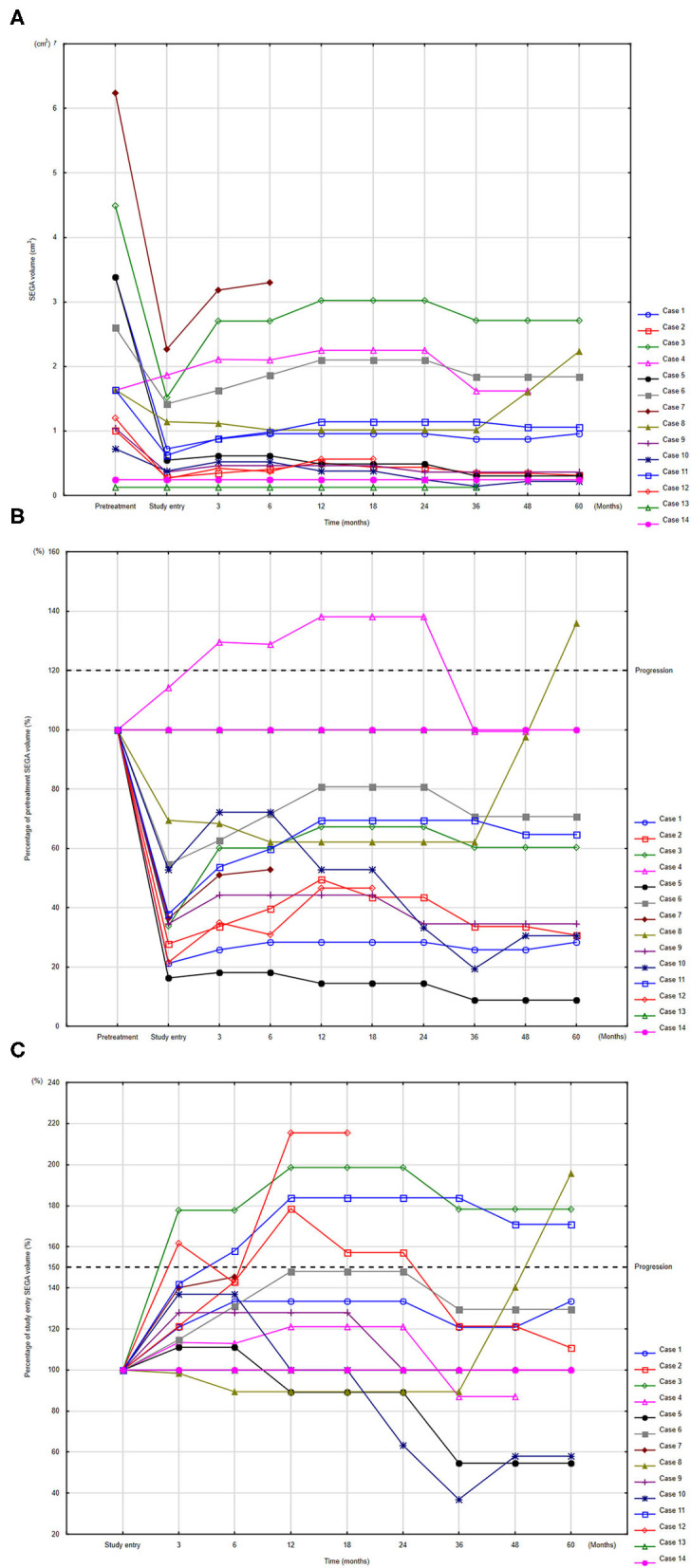
Changes in SEGA volume (SV) compared to volume before everolimus treatment **(A)**, and percentages of SV change at study time point compared to pretreatment measurements **(B)** and to study entry **(C)**. To identify respective individuals, the indices for each patient are consistent among panels **(A–C)**. Progression defined according to RECIST **(B)** and according to EMINENTS study **(C)**.

The changes in SEGA volumes in individual patients are presented in Figures 2A–C. Seven patients demonstrated progression (Supplementary Table 2). Of these, three patients discontinued the study. One patient underwent neurosurgery after 6 months of maintenance therapy due to enlargement of ventricular volume and risk of hydrocephalus. This patient demonstrated the greatest SV of the group, which increased during the study from 2.27 cm^3^ to 3.3 cm^3^ (145%); however, the patient did not exactly meet the progression criteria. In this case, mean everolimus concentration during the study was extremely low 0.57 ng/ml (95%CI: 0.16–1.99) (Supplementary Table 2) indicating poor compliance with everolimus therapy. In the two other patients, everolimus was escalated to daily treatment (full dose treatment).

The remaining four patients with progression continued the study with a reduced dose of everolimus; in two of these, SV decreased and they met the stabilization criteria. No clinical symptoms of progression were observed in any patients.

The patients who demonstrated stable SV during the whole study are compared with those presenting progression at any time in Table 2. No statistically significant differences were observed between groups with regard to in age, sex, TSC status, neurological status, pretreatment SV, SV at study entry, or in everolimus concentration and everolimus dose at study entry or during the study.

**Table 2:** Comparison of patients with stable SEGA volume (SV) vs. patients with progression of SEGA volume at any time during the study (two-tailed Fischer's exact test, UMW test according to distribution of the data).

**Characteristic**	**Patients with stabilization** **of SV (*n* = 7)**	**Patients with progression** **of SV (*n* = 7)**	***p*-value**
Age (years)	14.08 (8.8–19.37)	13.56 (11.14–15.98)	0.46
Sex			1
•Male	4	5	
•Female	3	2	
TSC status			0.76
•TSC1	2	2	
•TSC2	4	4	
•No mutation	1	1	
TSC-associated neuropsychiatric disorders	5/7	5/7	1
Epilepsy	5/7	6/7	1
SV before everolimus treatment (cm^3^)	1.64(0.31–2.98)	2.55 (0.7–4.41)	0.32
SV at study entry (cm^3^)	0.54 (0.14–0.94)	1.14 (0.41–1.86)	0.16
Percentage of SV at study entry compared to pretreatment (%)	54.23 (22.41–86.04)	48.72 (18.58–78.86)	1
Everolimus concentration at study entry (ng/ml)	8.53 (5.67–11.4)	8.10 (5.2–11)	0.8
Everolimus concentration during the study (ng/ml)	2.93 (2.03–3.82)	2.37 (1.53–3.21)	0.38
Everolimus dose at study entry (mg/m^2^/week)	43.06 (30.48–55.64)	38.93 (29.52–48.34)	0.62
Everolimus dose at study end (mg/m^2^/week)	16.02 (12.22–19.83)	14.71 (10.15–19.27)	0.53
Treatment decision			–
•continuation of MT	7	4^*^	
•neurosurgery^a^	0	1	
•full-dose treatment^b^^,^^c^	0	2	

* Four patients with SV progression continued the study; of these, two patients with SV met stabilization criteria after 24 months (one patient) and 30 months (one patient) of maintenance dose. Three patients discontinued the study:

a one patient - neurosurgical intervention;

b one patient – returned to full-dose treatment due to progression of SV (parents' decision);

c *one patient – returned to full-dose treatment due to progression of SV (investigator's decision)*.

The other TSC lesions observed in the study group are presented in Table 1. The study safety concerns indicate that any of coexisting features did not deteriorate significantly during the study.

### Adverse Events

Adverse events related to everolimus therapy are presented in Table 3.

**Table 3:** Adverse events related to everolimus in the study group during standard and maintenance therapy. NS, not statistically significant (*p* > 0.05) in a two-tailed Fisher's exact test.

	**Standard therapy**		**Maintenance therapy**										***p*-value**
			**1^st^year**		**2^nd^year**		**3^rd^year**		**4^th^year**		**5^th^year**		
**Adverse event**	**All grade**	**Grade 3**	**All grade**	**Grade3**	**All grade**	**Grade 3**	**All grade**	**Grade 3**	**All grade**	**Grade 3**	**All grade**	**Grade 3**	
**Infectious adverse events**													
Stomatitis	9/14	0	2/14	0	1/12	0	4/12	0	2/11	0	2/10	0	0.001
Pharyngitis	8/14	0	6/14	0	5/12	0	6/12	0	5/11	0	2/10	0	0.25
Bronchitis	6/14	0	1/14	0	0	0	0	0	1/11	0	0	0	0.03
Diarrhea	6/14	0	0	0	0	0	1/12	0	0	0	0	0	0.0007
Skin/soft tissue infection	4/14	1/14	0	0	0	0	0	0	1/11	0	0	0	0.55
Otitis (media/externa)	1/14	0	0	0	0	0	0	0	0	0	1/10	0	0.55
Sinusitis	0	0	0	0	0	0	0	0	1/11	0	0	0	−
Urinary tract infection	1/14	0	0	0	1/12	0	1/12	0	0	0	2/10	0	0.21
Vulval infection	1/5	0	2/5	0	0	0	0	0	0	0	0	0	0.42
Infections - total	11/14	1/14	7/14	0	5/12	0	6/12	0	6/11	0	4/10	0	0.04
Infections other than pharyngitis	7/14	1/14	2/14	0	0/12	0	3/12	0	4/11	0	2/10	0	0.005
**Other clinical/ Non-infectious adverse events**													
Irregular menses	3/5 girls	0	2/4 girls	0	2/4 girls	0	2/4 girls	0	2/4 girls	0	2/4 girls	0	−
Hypertension	3/14	0	2/14	0	2/12	0	2/12	30	2/11	0	2/11	0	0.42
Sinus tachycardia	1/14	0	0	0	0	0	0	0	0	0	0	0	0.42
Constipation	1/14	0	1/14	0	1/10	0	0	0	0	0	0	0	0.42
**Laboratory abnormality**													
Hypercholesterolemia	9/14	0	10/14	0	9/12	0	6/12	0	7/11	0	7/10	0	0.19
Hypertriglyceridemia	9/14	0	7/14	0	9/12	0	5/12	0	6/11	0	6/10	0	0.31
Neutropenia	7/14	1/10	0	0	2/12	0	0	0	0	0	0	0	0.001
Anemia	6/14	0	2/14	0	3/12	0	2/12	0	1/11	0	1/10	0	0.05
Hyperglycemia	4/14	0	0	0	0	0	0	0	0	0	0	0	0.01
Gamma-glutamyltransferase increased	3/14	1/10	0	0	0	0	0	0	0	0	0	0	−
Leucopenia	2/14	0	0	0	2/12	0	0	0	0	0	0	0	0.16
Alanine/Aspartate aminotransferase increased	2/14	0	1/14	1/13	1/12	0	0	0	0	0	0	0	0.35
Thrombocytopenia	1/14	0	1/14	0	3/12	0	1/12	0	1/11	0	0	0	0.23
Bilirubin increased	1/14	0	1/14	0	2/12	0	2/12	0	1/11	0	1/10	0	0.42

AEs were divided into two groups: clinical AEs and laboratory abnormalities. Clinical AEs were observed in 11/14 patients during maintenance therapy. The most common were the infections which led to dose interruptions (9/14 patients). No grade three or four clinical AEs were noted. Clinical AEs related to everolimus administration did not lead to cessation of treatment in any patient.

Laboratory abnormalities were recorded in 13/14 patients during the maintenance dose. They were of mild or moderate severity (grade one or two) and they did not lead to dose interruption or cessation of treatment in any patient.

Of the clinical AEs, infections in total occurred less frequently during maintenance therapy compared to the standard dose regimen, with the most common ones being stomatitis, bronchitis and diarrhea. The same dependency was noted for some laboratory abnormalities, in particular neutropenia, anemia and hyperglycemia (Table 3).

## Discussion

This is the final report of the EMINENTS trial, which has previously been reported as an interim analysis for shorter follow-up for only 10 patients (10). Long-term maintenance therapy resulted in an insignificant increase of SEGA volume. Of 14 analyzed patients, eleven continued the study; of these, seven demonstrated stabilization of tumor volume during the whole observation period while the other four met the criteria of progression at some point in the study. Of those, four patients, who continued the reduced everolimus dosing regimen despite progression, SEGA volume decreased in two patients, meeting the stabilization criteria after a subsequent 24 and 30 months of maintenance therapy, while tumor size remained stable for the remainder of the therapy in the other two. No symptoms of progression were observed in any patient. Despite seven out of 14 patients experienced progression of SEGA, 11 patients (78%) continued a maintenance dose with subsequent stabilization or shrinkage of the tumor. These findings indicate that the continuation of a maintenance dose, even in case of growing SEGA, does not rule out a final good treatment effect in those patients under close clinical and imaging monitoring. As no differences in clinical characteristics were found between patients with progression and those who demonstrated stabilization of tumor volume, no risk factors of progression could be identified. In our study progression was defined as a tumor regrow comparing to the volume at study entry, which was a residual mass after 1 year of full dose everolimus therapy. This do not mean the same as progression used in oncology which is an increase of primary tumor volume.

Three patients discontinued the study. One patient underwent neurosurgery after 5 months of maintenance therapy with everolimus due to enlargement of ventricular volume and risk of hydrocephalus. In two patients, the everolimus dose was escalated to full dose treatment: this change was the investigator's decision in one patient, and the parents' decision in the second, i.e., the parents withdrew their consent to participate in the study.

To summarize, of the three patients who discontinued the study, only one truly required dose escalation related to progression of SV after 60 months of MT: the other two left due to noncompliance in one case and withdrawal of consent in the other. However, even in this case with “true progression,” no clinical symptoms of SEGA growth were noted and no signs of hydrocephalus were visible in the MRI.

SV progression has also been observed in other studies during full dose everolimus treatment: in 11.7% of patients in the EXIST-1 Study and 0.8% of patients in the EFECTS (6, 14). The SEGA regrowth was also observed in case series study by Weidman et al. in two out of four patients after cessation or reduction of sirolimus. In this limited series doses < 2.5 mg/m^2^ were insufficient to maintain SEGA response (15). In our study, average dose of everolimus maintenance treatment was 15.37 mg/m^2^/week (2.2 mg/m^2^/day), showing therapeutic effect in 11 out of 14 patients. However, doses between 2 and 3 mg/m^2^ may represent a gray zone where SEGA regrowth might be observed in some patients (15). A dose-response relation might be connected to the volume of the tumor, meaning that bigger volume needs higher dose. But this is only a hypothesis and the optimal dose of everolimus to maintain SEGA response requires further research.

Mean everolimus concentration was significantly lower during the study than at study entry (2.65ng/ml; 95%CI: 2.1 – 3.19 vs. 8.32ng/ml; 95%CI: 6.58 – 10.05). However, there were no differences in mean everolimus concentration between the patients demonstrating stabilization of SV during the whole study and those with progression at any time of the study. Final analysis of EXIST-Study reported that responses occurred despite a majority of patients had a median serum level of everolimus below or just within the usual therapeutic range. The authors stated that efficacy et a lower serum level may result in fewer adverse effects and better tolerability (5). In our previous report, no significant differences in SV reduction were observed between patients with everolimus trough level < 5ng/ml and those with levels ≥ 5ng/ml, suggesting that drug dose titration according to blood concentration does not play a key role in achieving clinical success in SEGA treatment (9).

Although adverse events are very commonly observed during MTOR inhibitor treatment in patients with TSC, they are usually mild or moderate in severity (16, 17); however, life-threating events also have been reported (8). The AEs reported in this study were less severe and significantly less common than those observed during the standard dose period; this difference was particularly apparent after the exclusion of pharyngitis, which is a common health problem in children independent of immune status. The most common AEs were stomatitis, bronchitis and diarrhea, which are common reasons of treatment interruption. A recent meta-analysis found stomatitis and upper respiratory tract infections to be the most commonly-reported AEs (18).

The most important observation of our study was that no SAE was reported during MT and none of the patients discontinued the study due to AEs. This is in contrast to observations made during full dose everolimus treatment. In the EFFECTS-study, SAE was reported in 26.7% patients and AEs led to study drug discontinuation in 6.7% patients (14). In the EXIST-1 study, almost 10% of patients experienced an AE that led to everolimus discontinuation (6).

The number of patients with TSC receiving everolimus treatment is steadily growing as the numbers of clinical manifestations of TSC as indications for MTOR inhibitors, either approved or under controlled clinical trials, are also increasing (19). In addition, such treatment may be long-term or perhaps indefinite. Hence, there is a greater need to consider the safety issues associated with treatment. It is possible that maintenance therapy with everolimus might represent a rational therapeutic option for this growing population after effective full dose treatment. It could be an option for patients who experienced everolimus-related AEs, instead of discontinuation of therapy.

The present analysis is limited by the open-label and single-arm nature of its design.

However, comparison made with previous full-dose everolimus treatment period in terms of SV and AEs allowed to draw significant conclusions.

Another limitation is the small number of patients recruited to the study. However, as only 40 children with TSC and SEGA were treated with everolimus in Poland during the study period, our data represent a significant part of this population.

Although the research was conducted as a single-center study, the center was one of the most experienced in Poland for treating SEGA related to TSC. Thus, the decision about reduction of therapy was made by an experienced team, after close consideration of the situations of both patients and parents. Careful evaluation of possible progression, especially concerning first six months of maintenance therapy should be advised.

## Conclusion

Maintenance therapy with everolimus might represent a rational therapeutic option for patients TSC and SEGA after effective full dose treatment, especially it could be an option for patients who experienced everolimus-related AEs, instead of discontinuation of therapy.

Our results suggest that progression of SEGA might be asymptomatic; in addition, no patient of tumor-related risk factors of progression could be identified. Therefore, close monitoring of SEGA volume on maintenance therapy should be recommended.

Continuation of a maintenance dose, even in case of slowly growing SEGA, does not rule out a final good treatment effect under strict clinical and imaging monitoring.

## Data Availability Statement

The raw data supporting the conclusions of this article will be made available by the authors, without undue reservation.

## Ethics Statement

The studies involving human participants were reviewed and approved by Komisja Bioetyczna przy Uniwersytecie Medycznym w Łodzi. Written informed consent to participate in this study was provided by the participants' legal guardian/next of kin.

## Author Contributions

KB, KKr, and WM (specialist of pediatric oncology) collected clinical data and wrote the draft of the manuscript and approved the final manuscript as submitted additionally. KB (specialist in statistic) performed statistical analysis. DB (specialist in radiology) evaluated MRI scans. KKa and SJ (specialist in neurology) provided some clinical and genetic data and contributed to writing of the manuscript and approved the final manuscript as submitted. JT designed the study and prepared the final version of the manuscript and approved the final manuscript as submitted. All authors contributed to the article and approved the submitted version.

## Conflict of Interest

The authors declare that the research was conducted in the absence of any commercial or financial relationships that could be construed as a potential conflict of interest.

## Abbreviations

SV: SEGA volume
AEs: adverse events.
