# Supplementary material for: Maintenance Therapy With Everolimus for Subependymal Giant Cell Astrocytoma in Patients With Tuberous Sclerosis – Final Results From the EMINENTS Study
Source: Front Neurol. 2021 Apr 9;12:581102. doi: 10.3389/fneur.2021.581102 (PMC8062974; doi:10.3389/fneur.2021.581102)
Supplement: Supplementary file 1 [file Table_1.DOCX]

Supplementary Table 1. The mean SEGA volume (SV), percentage of SV compared with pretreatment measurements and percentage of SV compared with volume at study entry - before standard treatment and at study time points by manual method, number of patients with stable SV and median everolimus concentration at study time points

| Time point | No of patients in the study | No (%) of patients with stable SV | Everolimus concentration (mean [95 CI for SD]) | p-level (ANOVA repeated measures, post-hoc unequal N HSD) comparing to study entry | | SV (cm^3^) (mean [95 CI for SD]) | p-level (ANOVA repeated measures, post-hoc unequal N HSD) comparing to | | percentage of SV compared to pretreatment (%) (mean [95 CI for SD]) | p-level (ANOVA repeated measures)=0.32 | percentage of SV compared to study entry (%) (mean [95 CI for SD]) | p-level (ANOVA repeated measures) =0.29 |
| --- | --- | --- | --- | --- | --- | --- | --- | --- | --- | --- | --- | --- |
|  |  |  |  | Study entry | 3 months |  | Pretreatment | Study entry |  |  |  |  |
| Pretreatment | 14 | - | - | - | - | 2.1 (1.26 - 2.8) | - | 0,000158 | - | - | - | - |
| Study entry | 14 | - | 8.32 (2.18 - 4.84) | - | 0.00014 | 0.84 (0.5 - 1.19) | 0,000158 | - | 51.47 (23.43 - 52.07) | - | - | - |
| 3 months | 14 | 11 (78,5) | 2.23 (1.1 - 2.45) | 0.00014 | - | 1.09 (0.7 - 1.55) | 0,000158 | 0.95 | 61 (22.8 - 50.67) | - | 126.1 (17.04 - 37.87) | - |
| 6 months^a^ | 14 | 10 (71) | 2.61 (1.37 - 3.04) | 0.00014 | 1,000000 | 1.12 (0.72 - 1.6) | 0,000158 | 0.88 | 62.05 (22.47 - 49.94) | - | 129.18 (17.72 - 39.37) | - |
| 12 months | 13 | 8 (62) | 2.55 (1.08 - 2.6) | 0.00014 | 0,999999 | 1.02 (0.64 - 1.48) | 0,000160 | 0.7 | 65.68 (23.7 - 54.56) | - | 137.31 (31.4 - 72.28) | - |
| 18 months^b^ | 13 | 8 (62) | 2.2 (0.86 - 1.98) | 0.00014 | 1,000000 | 1.01 (0.64 - 1.48) | 0,000160 | 0.72 | 65.22 (23.9 - 55.02) | - | 135.66 (30.47 - 70.14) | - |
| 24 months | 12 | 8 (67) | 2.86 (1.39 - 3.34) | 0.00014 | 0,973917 | 1.03 (0.67 - 1.6) | 0,000159 | 0.81 | 64.35 (25.57 - 61.3) | - | 123.63 (29.26 - 70.13) | - |
| 36 months | 12 | 10 (83) | 2.4 (1 - 2.39) | 0.00014 | 0,999701 | 0.9 (0.58 - 1.39) | 0,000158 | 0.99 | 57.05 (23.03 - 55.2) | - | 108.5 (30.5 -73.11) | - |
| 48 months | 11 | 9 (82) | 2.56 (0.94 - 2.5) | 0.00014 | 0,999929 | 1.01 (0.58 - 1.46) | 0,000158 | 0.93 | 56.92 (22.74 - 57.1) | - | 114.63 (28.13 - 70.66) | - |
| 60 months^c^ | 10 | 7 (70) | 3.9 (1.11 - 3.14) | 0.00014 | 0,583891 | 1.02 (0.64 - 1.69) | 0,000160 | 0.73 | 56.49 (26.51 - 70.36) | - | 123.09 (33.16 - 88) | - |

Three patients discontinued the study: ^a^ one patient - neurosurgical intervention; ^b^ one patient – returned to full-dose treatment due to progression of SV (parents’ decision); ^c^  one patient – returned to full-dose treatment due to progression of SV (investigator’s decision);

Supplementary Table 2. The clinical characteristics of the patients with progression of SEGA volume at any time of the study

|  | Patient CzB (Case 2) | Patient LN (Case 3) | Patient GJ (Case 4) | Patient KM (Case 7) | Patient FF (Case 8) | Patient KK (Case 11) | Patient MJ (Case 12) |
| --- | --- | --- | --- | --- | --- | --- | --- |
| Age (years) | 13.4 | 11.5 | 13.3 | 16.5 | 9.5 | 16.9 | 13.9 |
| Sex | M | F | M | F | M | M | M |
| TSC status | TSC1 | TSC2 | TSC2 | TSC2 | No mutation | TSC2 | TSC1 |
| SV before ET (cm^3^) | 1.01 | 4.49 | 1.63 | 6.24 | 1.64 | 1.64 | 1.2 |
| SV at study entry (cm^3^) | 0.28 | 1.52 | 1.86 | 2.27 | 1.14 | 0.62 | 0.26 |
| Percentage of SV at study entry compared to pretreatment (%) | 28 | 34 | 114 | 36 | 69 | 38 | 22 |
| Progression criterion | SV>150% SV at study entry | SV>150% SV at study entry | SV> pretreatment SV | Enlargement in ventricular volume | SV>150% SV at study entry | SV>150% SV at study entry | SV>150% SV at study entry |
| SV at progression (cm^3^) | 0.5  (1.1x0.8x1.1) | 2.7  (1.6x1.8x1.8) | 2.11  (1.5x1.5x1.8) | 3.3  (2.2x1.6x1.8) | 2.23  (1.8x1.4x1.7) | 0.96  (1.2x1.4x1.1) | 0.56  (0.9x1.0x1.2) |
| SV at the study end (cm^3^) | 0.31 | 2.7 | 1.62 | 3.3 | 2.23 | 1.06 | 0.56 |
| Time-point of progression | 12 months | 3 months | 3 months | 6 months | 60 months | 6 months | 3 months |
| Everolimus concentration at study entry (ng/ml) | 11.29 | 7.87 | 11.6 | 2.3 | 6.56 | 8.4 | 8.7 |
| Everolimus concentration at progression (ng/ml) | 3.3 | 0.9 | 4.6 | 0.5 | 0.9 | 2.9 | 1.4 |
| Everolimus concentration during the study (ng/ml) | 3.3+/-1.61 | 2.06+/-1.9 | 2.88+/-2.34 | 0.57+/-0.32 | 2.67+/-2.27 | 2.14+/-1.02 | 2.8+/-1.3 |
| Clinical symptoms at progression | none | none | none | none | none | none | none |
| Treatment decision | continuation of MT | continuation of MT | continuation of MT | Neurosurgery.  No compliance | full dose treatment (investigator’s decision) | continuation of MT | full dose treatment  (parents’ decision) |
| Outcome after intervention | SV decreasing Stabilization after subsequent 24 months of MT | SV stable  Maintained progression criteria | SV decreasing Stabilization after subsequent 30 months of MT | No SEGA | SV stable  Maintained progression criteria | SV stable  Maintained progression criteria | SV stable  Maintained progression criteria |

To identify respective individuals, the numbers for each patient are consistent with Figure 2
